# Downregulation of miR-137 and miR-6500-3p promotes cell proliferation in pediatric high-grade gliomas

**DOI:** 10.18632/oncotarget.7736

**Published:** 2016-02-25

**Authors:** Muh-Lii Liang, Tsung-Han Hsieh, Kim-Hai Ng, Ya-Ni Tsai, Cheng-Fong Tsai, Meng-En Chao, Da-Jung Liu, Shing-Shiung Chu, Wan Chen, Yun-Ru Liu, Ren-Shyan Liu, Shih-Chieh Lin, Donald Ming-Tak Ho, Tai-Tong Wong, Muh-Hwa Yang, Hsei-Wei Wang

**Affiliations:** ^1^ Institutes of Clinical Medicine, National Yang-Ming University, Taipei, Taiwan; ^2^ Division of Pediatric Neurosurgery, Neurological Institute, Taipei Veterans General Hospital, Taipei, Taiwan; ^3^ PhD Program for Cancer Biology and Drug Discovery, College of Medical Science and Technology, Taipei Medical University, Taipei, Taiwan; ^4^ Comprehensive Cancer Center of Taipei Medical University, Taipei Medical University, Taipei, Taiwan; ^5^ Institute of Microbiology and Immunology, National Yang-Ming University, Taipei, Taiwan; ^6^ Institute of Biomedical Informatics, National Yang-Ming University, Taipei, Taiwan; ^7^ Institutes of Clinical Medicine, Taipei Medical University, Taipei, Taiwan; ^8^ Department of Neurosurgery, Taipei Medical University Hospital, Taipei Medical University, Taipei, Taiwan; ^9^ Joint Biobank, Office of Human Research, Taipei Medical University, Taipei, Taiwan; ^10^ School of Medicine, National Yang-Ming University, Taipei, Taiwan; ^11^ National PET/Cyclotron Center, Department of Nuclear Medicine, Taipei Veterans General Hospital, Taipei, Taiwan; ^12^ Molecular and Genetic Imaging Core/Taiwan Mouse Clinic National Comprehensive Mouse Phenotyping and Drug Testing Center, Taipei, Taiwan; ^13^ Department of Pathology and Laboratory Medicine, Taipei Veterans General Hospital, Taipei, Taiwan; ^14^ Cancer Research Center & Genome Research Center, National Yang-Ming University, Taipei, Taiwan; ^15^ Division of Hematology-Oncology, Department of Medicine, Taipei Veterans General Hospital, Taipei, Taiwan; ^16^ Immunity and Inflammation Research Center, National Yang-Ming University, Taipei, Taiwan; ^17^ Genomic Research Center, Academia Sinica, Taipei, Taiwan

**Keywords:** miR-137, miR-6500-3p, CENPE, KIF14, NCAPG

## Abstract

Pediatric high-grade gliomas (pHGGs) are aggressive brain tumors affecting children, and outcomes have remained dismal, even with access to new multimodal therapies. In this study, we compared the miRNomes and transcriptomes of pediatric low- (pLGGs) and high-grade gliomas (pHGGs) using small RNA sequencing (smRNA-Seq) and gene expression microarray, respectively. Through integrated bioinformatics analyses and experimental validation, we identified miR-137 and miR-6500-3p as significantly downregulated in pHGGs. miR-137 or miR-6500-3p overexpression reduced cell proliferation in two pHGG cell lines, SF188 and UW479. CENPE, KIF14 and NCAPG levels were significantly higher in pHGGs than pLGGs, and were direct targets of miR-137 or miR-6500-3p. Furthermore, knockdown of CENPE, KIF14 or NCAPG combined with temozolomide treatment resulted in a combined suppressive effect on pHGG cell proliferation. In summary, our results identify novel mRNA/miRNA interactions that contribute to pediatric glioma malignancy and represent potential targets for the development of new therapeutic strategies.

## INTRODUCTION

Gliomas are a heterogeneous group of malignant neoplasms that are thought to arise from glial progenitor cells and astrocytes [1]. They are classified based on the following criteria: increased cellular density, nuclear atypia, mitosis, vascular proliferation and necrosis. The World Health Organization (WHO) classifies pediatric gliomas, the most common pediatric central nervous system tumors, into grades I through IV [2, 3]. Pediatric low-grade gliomas (pLGGs) (grades I and II) account for 30% of all pediatric brain tumors and have a favorable prognosis [2, 4, 5]. However, pediatric high-grade gliomas (pHGGs) (grades III and IV), which account for only 8-10% of all pediatric brain tumors, grow faster and are invasive; this hinders total resection, especially in the case of glioblastoma multiforme (GBM) [2, 5, 6]. Following maximal safe surgical resection of pHGGs, adjuvant fractionated radiotherapy is usually applied in children over three years of age. Chemotherapy (CCG-943, lomustine, vincristine and prednisone) combined with radiation therapy has been demonstrated to prolong survival compared to radiation treatment alone (48% *vs*. 15%, 5 year progression-free survival) [5], but overall pHGG patient survival rates remain dismal. More recently, concurrent oral administration of temozolomide (TMZ) with radiation has become the first-line treatment for newly diagnosed pHGG patients in many institutions [7]. Still, infiltrating malignant cells that survive initial treatments remain the most challenging issue.

Genetic profiling has provided insight into differences between pHGGs and adult HGGs over the last decade. *PTEN* alterations, loss of 10q, gain of chromosome 7, *EGFR* and *CDKN2A* amplifications and hotspot mutations in *IDH1* are less common in pHGGs than in adult HGGs [8-10], while gain of 1q and loss of 1p, 4q, and 16q is more common in pHGGs [9]. *PDGFRA* is also amplified in pHGGs, especially in irradiation-induced tumors [8]. These findings suggest that the mechanisms associated with malignant gliomagenesis in children are different from those in adults.

MicroRNAs (miRNAs) play important roles in the modulation of tumorigenesis. A previous study showed the presence of 23 upregulated and 17 downregulated miRNAs in pediatric gliomas compared to normal brain tissue [11]. These 40 differentially expressed miRNAs regulate gene expression of tumor- and nervous system-related pathways. Miele,*et al.* showed that the miR-17∼92 cluster is overexpressed in pHGGs compared to adult HGGs and targets the important tumor suppressor genes, *PTEN* and *RB1* [12].

In the present study, we analyzed the miRNomes and transcriptomes of pLGGs and pHGGs using small RNA high-throughput sequencing (smRNA-seq) and gene expression microarray analysis, respectively. We found that two miRNAs downregulated in pHGGs, miR-137 and miR-6500-3p,decreased proliferation of pHGG cells. We identified three genes, *KIF14*, *NCAPG* and *CENPE* that were upregulated in pHGGs and were direct miR-137 or miR-6500-3p targets. *CENPE*, *KIF14* or *NCAPG* knockdown combined with TMZ treatment suppressed proliferation in pHGG cells. Our findings provide potential new targets for the development of improved therapeutic strategies.

## RESULTS

### Clinical characterization of the pediatric gliomas

**Figure 1:**
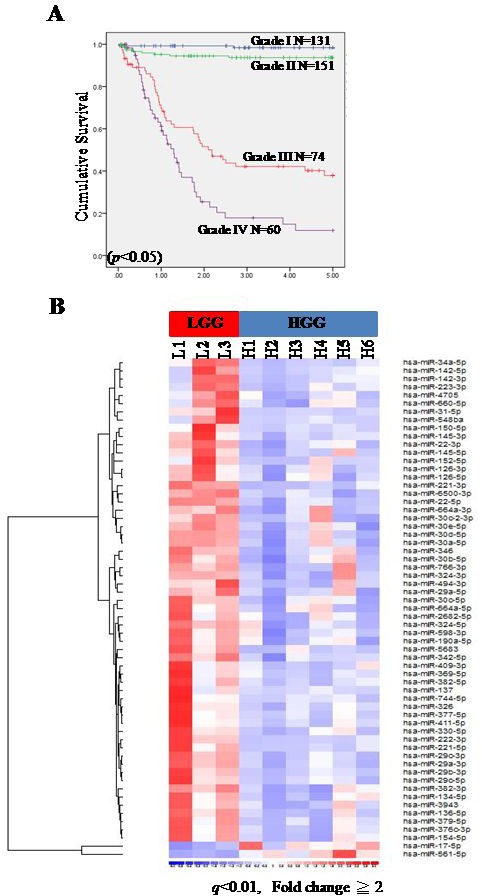
Small RNA sequencing (smRNA-seq) analysis of pediatric gliomas Five-year Kaplan-Meier survival curves for pediatric glioma patients at Taipei Veterans General Hospital from 1971-2013 **A.** Overall survival time was calculated as the time from diagnosis to death or to last follow-up. Blue line: grade I (LGG); green line: grade II (LGG); red line: grade III (HGG); purple line: grade IV (HGG). A heat map illustrating differentially expressed miRNAs between pLGGs (*n* = 3) and pHGGs (*n* = 6) **B.** A total of 59 miRNAs were upregulated in pLGGs and 2 were upregulated in pHGGs.

We reviewed a total of 416 pediatric glioma cases that had been diagnosed and treated at Taipei Veterans General Hospital between 1971 and 2013(Figure S1A & S1B). About two-thirds (282/416, 67.8%) were pLGGs and about one-third (134/416, 32.2%) were pHGGs. Pilocytic astrocytomas (125/282, 44.3%) and anaplastic astrocytomas (68/134, 50.7%) were the most common tumor types among pLGGs and pHGGs, respectively. The mean age of pLGG patients was similar to that of pHGG patients (7.7 *vs*. 8.9 years old) (Table 1). Overall, pHGG patients (grades III & IV) had poorer overall survival than pLGG patients (grades I & II) using Kaplan-Meier survival curves (Figure 1A and S1C). The 2- and 5-year overall survival rates were 99.2% and 98.5%, 94.7% and 94.0%, 55.4% and 44.6% and 38.3% and 30.0% for grade I, II, III and IV gliomas, respectively (*p* < 0.05).

**Table 1:** Overview of the pediatric patients with LGG and HGG

	LGG	HGG
Number	282	134
Age		
< 3 years	55	22
3-10 years	129	54
> 10 years	98	58
mean	7.7 years	8.9 years
range	1 days −18.8 years	10 days −18.7 years
Gender		
male	146	71
female	136	63

### miR-137 and miR-6500-3p downregulation in pHGGs increased cell proliferation

To identify miRNome differences between pLGG and pHGG, the global miRNA expression patterns of three pLGGs and six pHGGs were investigated by small RNA sequencing (smRNA-Seq) [13]. We identified 61 known miRNAs as differentially expressed between pLGGs and pHGGs (*p* < 0.05; Reads per kilo base of exon model per million mapped reads (RPKM)≥2 in at least 2/3 pLGG and pHGG cases) (Figure 1B, Table S1). In particular, miR-137 andmiR-6500-3p were most significantly downregulated in pHGGs

**Figure 2:**
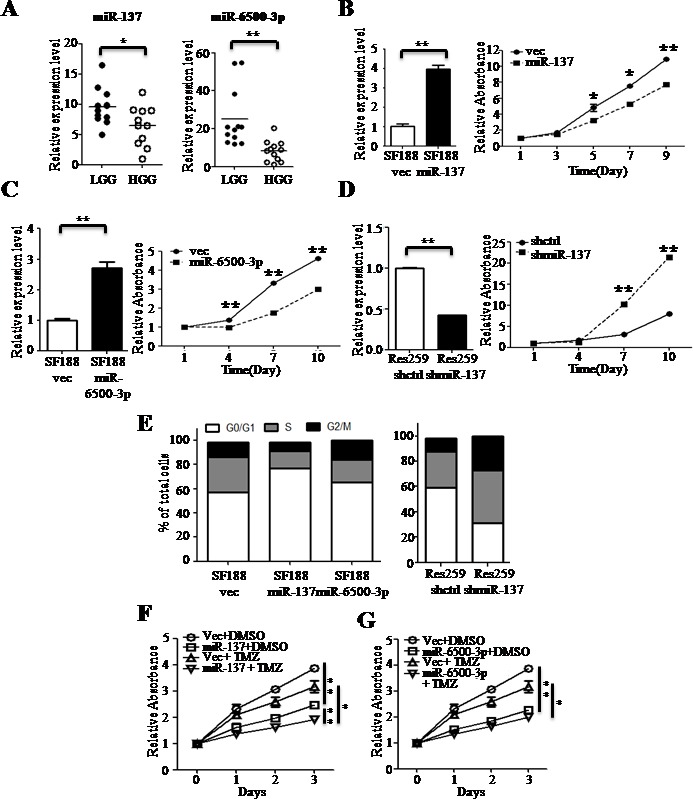
miR-137 and miR-6500-3p suppress cell proliferation and have a combined effect with TMZ treatment RT-qPCR confirmed downregulation of miR-137 and miR-6500-3p expression in pHGGs (*n* = 12) compared to pLGGs (*n* = 12) **A.** Overexpression of miR-137 **B.** or miR-6500-3p **C.** decreases cell proliferation in SF188 cells, and miR-137 knockdown **D.** increases cell proliferation in Res259 cells as measured by MTT assay. The effects of overexpression were evaluated by RT-qPCR. The effects of miR-137 or miR-6500-3p overexpression on SF188 cells (*left panel*) and of miR-137 knockdown on Res259 cells (*right panel*) were evaluated by flow cytometry **E.** SF188 cells overexpressing miR-137 **F.** or miR-6500-3p **G.** were treated with either DMSO or TMZ and cell proliferation was measured at different time points by MTT assay. Results are presented as mean±SD for duplicate samples. *p < 0.05, **p < 0.01 by *t*-test.

pHGGs have higher mitotic activity than pLGGs [6]. miR-137 was shown to decrease tumor proliferation in various cancers [14-16], and could act as a tumor suppressor. Prior to our study, the biological function of miR-6500-3p was unknown. We investigated the roles of miR-137 and miR-6500-3p in the regulation of cell proliferation in the pHGG cell lines, SF188 and UW379. We found that miR-137 and miR-6500-3p had the lowest expression ratio between pHGGs and pLGGs (pHGG/pLGG) in 24 clinical samples (Figure 2A). Overexpression of miR-137 ormiR-6500-3p decreased cell proliferation in transfected cells (Figure 2B-2C and S2A-B). Knockdown of miR-137 in Res259 cells, a pLGG cell line, increased cell proliferation rates (Figure 2D). We also found that miR-137 or miR-6500-3p overexpression increased accumulation of the G1 phase cell fraction (Figure 2E and S2C), while miR-137 knockdown in Res259 cells resulted in a decreased G1 fraction.

Temozolomide (TMZ) is a DNA alkylating agent that can cross the blood-brain barrier (BBB). Clinically, TMZ had been demonstrated to be active against gliomas and to prolong overall survival rates of adult patients [17]. Previous study of pediatric malignant gliomas showed improved progression-free survival in patients with low tumor levels of MGMT receiving alkylation-based chemotherapy (97 out of 109 patients) [18]. MGMT is an O^6^-methylguanine DNA methyltransferase that removes methyl groups and repairs DNA adducts [17]. We found that overexpression of either miR-137 or miR-6500-3p combined with TMZ treatment decreased cell proliferation rates as compared to control cells treated with TMZ and cells overexpressing miR-137 or miR-6500-3p without TMZ treatment (Figure 2F-2G and S2D-E).

### Differentially expressed genes between pLGGs and pHGGs

**Figure 3:**
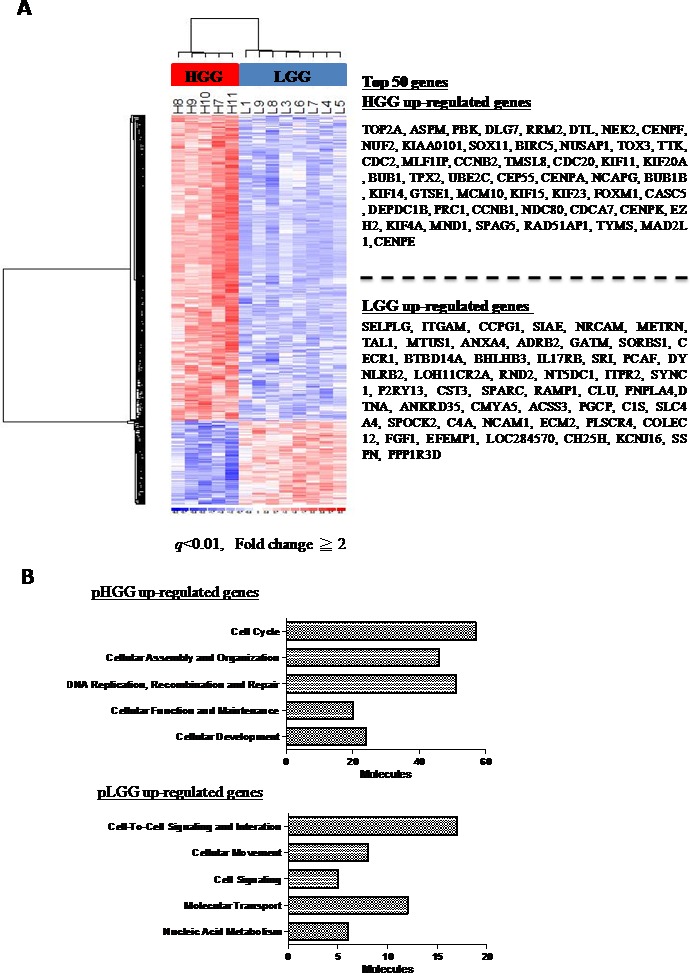
Gene expression microarray analysis of pediatric gliomas Heat map illustrating the expression of 457 probe sets (*q* < 0.01, fold change≥2) in five pHGGs and eight pLGGs, with the top 50 genes listed in the right panel **A.** 264 genes are overexpressed in pHGGs **B.** and 76 genes are overexpressed in pLGGs **C.** Genes overexpressed in pHGGs and pLGGs were subjected to Gene Ontology (GO) database searches.

We explored the differential molecular expression profiles of eight pLGGs and five pHGGs by microarray analysis and found that each type of glioma has unique transcriptome characteristics. A total of 360 probe sets (264 genes) were increased in pHGGs, while another 96 probe sets (76 genes) were increased in LGGs (*q* < 0.01, Fold change≥2, Figure 3A, Table S2). Gene Ontology analysis showed that most upregulated genes were involved in regulation of the cell cycle (57 genes) or DNA replication/repair (51 genes) (Figure 3B). In contrast, most upregulated genes in pLGGs were involved in cell-to-cell signaling/interaction (17 genes) or molecular transport (12 genes) (Figure 3C).

### Identifying the pLGG and pHGG miRNA-mRNA interactomes

**Figure 4:**
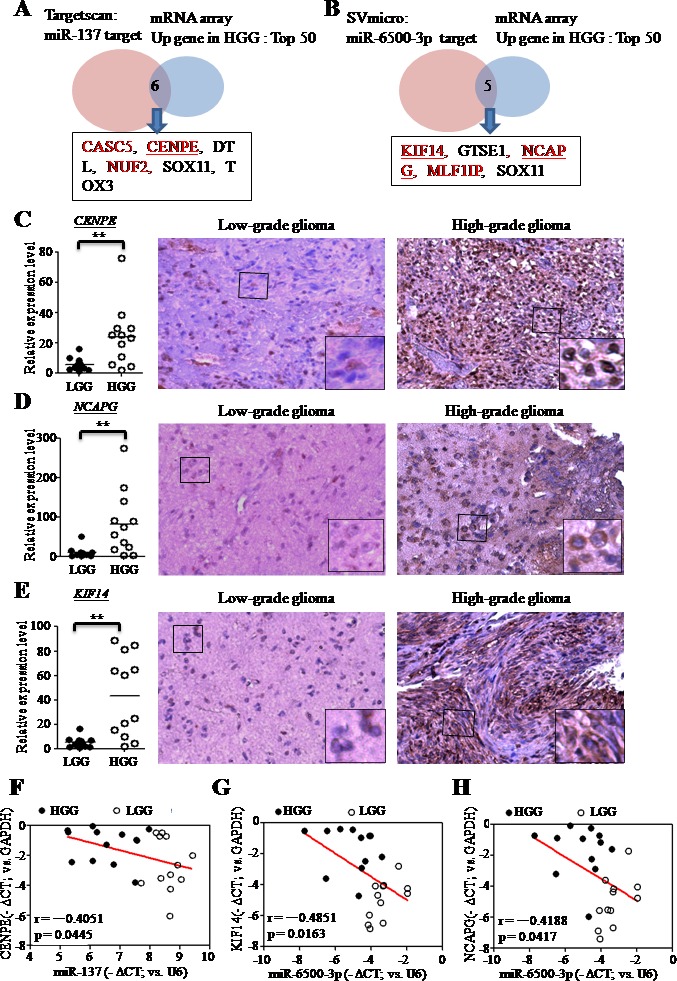
miR-137 and miR-6500-3p target *CENPE, KIF14* or *NCAPG*, which are upregulated in pHGGs Venn diagram showing overlapping mRNAs based on microarray data (top 50 upregulated mRNAs in pHGGs) and bioinformatics prediction results (Targetscan or SVmicro). The bioinformatic analysis indicates the miR-137 **A.** and miR-6500-3p **B.** targets. RT-qPCR and IHC analyses confirmed higher mRNA and protein levels of CENPE **C.**, KIF14 **D.** and NCAPG **E.** in pHGGs (mRNA: *n* = 12; protein: *n* = 3) than in pLGGs (mRNA: *n* = 12; protein: *n* = 3). RT-qPCR results are presented as mean±SD for duplicate samples. **p < 0.01 by t-test. Scatter plots illustrating the negative correlations between miR-137 and CENPE **F.**, miR-6500-3p and KIF14 **G.**, and miR-6500-3p and NCAPG **H.** in pediatric gliomas.

We used Svmicro and Targetscan (http://www.targetscan.org/) to identify which expressed genes were direct targets of miR-137 and miR-6500-3p, and compared these results with the top 50 upregulated genes in pHGGs. We found that miR-137 and miR-6500-3p targeted six (*CASC5*, *CENPE*, *DTL*, *NUF2*, *SOX11* and *TOX3*) and five (*KIF14*, *GTSE1*, *NCAPG*, *MLF1IP*, and *SOX11*) genes based on Targetscan and SVmicro prediction, respectively (Figure 4A-4B). Gene ontology showed that *CASC5*, *CENPE*, *NUF2*, *KIF14*, *NCAPG* and *MLF1IP* were cell-cycle related genes.

mRNA and protein expression levels of all genes were assessed using qRT-PCR and IHC staining, which showed that *CENPE*, *KIF14* and *NCAPG* were potential targets of miR-137 and miR-6500-3p. *CENPE* (centromere-associated protein-E) is a kinesin-like centromere protein that is highly expressed during mitosis and is required for efficient and stable microtubule capture at the kinetochore [19, 20]. *KIF14* (kinesin family member 14) is a kinesin superfamily protein that plays an important role in regulation of the cell cycle and mitotic progression [21]. *NCAPG* (non-SMC condensin I complex subunit G) is a subunit of the condensin I complex, which plays important roles in meiotic chromosome segregation [22]. mRNA and protein levels of these three genes were increased in pHGG samples (Figure 4C-4E). We re-analyzed other microarray datasets [23] and found that levels of these three genes were also higher in pHGGs compared to pLGGs and normal brain tissue (Figure S3A-D). Analysis in prognoscan (http://www.prognoscan.org/) showed that overexpression of these three genes was associated with poor prognosis (Figure 3E-3G). Correlation coefficients (Pearson's *r*) of clinical samples indicated negative correlations between *CENPE* and miR-137 expression (−0.4051), *KIF14* and miR-6500-3p expression (−0.4851), and *NCAPG* and miR-6500-3p expression (−0.4188) (Figure 4F-4G).

**Figure 5:**
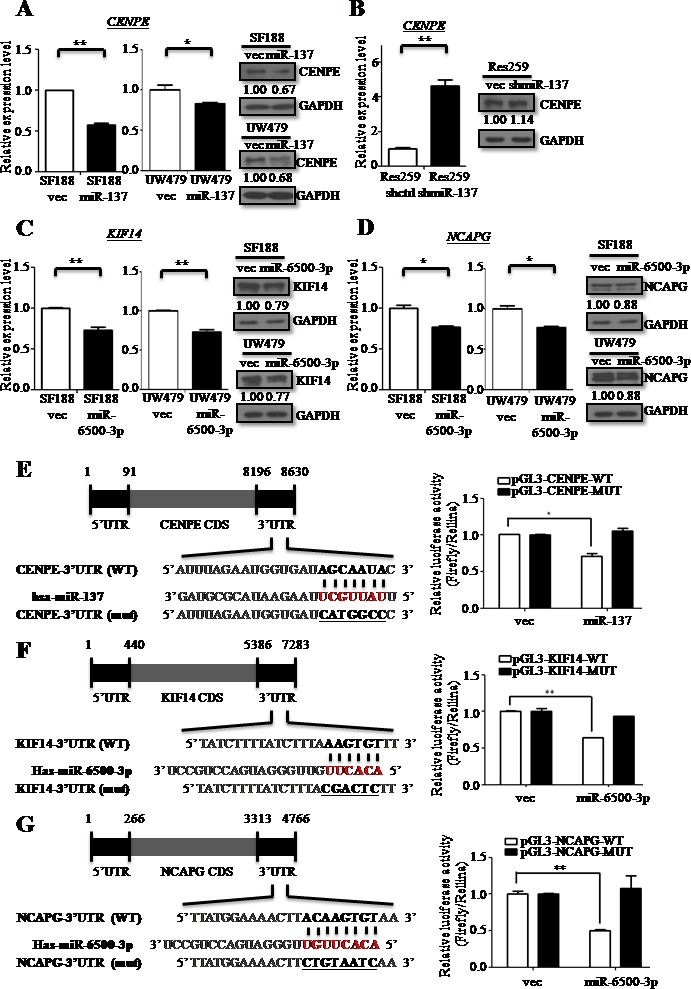
miR-137 and miR-6500-3p target directly *CENPE, KIF14* or *NCAPG* RT-qPCR and immunoblotting confirmed levels of CENPE **A.-B.**, KIF14 **C.** or NCAPG **D.** in SF188 and UW479 cells overexpressing miR-137 **A.** or miR-6500-3p **C.-D.**, and in Res259 cells with knocked-down miR-137 **B.** The predicted seed region duplexes formed between CENPE and miR-137 **E.**(*left panel*), KIF14 and miR-6500-3p **F.**(*left panel*), and NCAPG and miR-6500-3p **G.**(*left panel*) are shown. The seed-binding regions with base substitutions in the mutant (Mut) constructs are underlined. The relative luciferase activities of miR-137 or miR-6500-3p, when co-transfected with either the wild type or mutant 3′UTR reporter plasmids (*right panels*), are shown. Data are presented as mean±SD. *p < 0.05 by *t*-test.

We overexpressed miR-137 and miR-6500-3p in SF188 and UW479 cells and knocked-down miR-137 expression in Res259 cells, and analyzed *CENPE*, *KIF14* and *NCAPG* mRNA and protein levels using qRT-PCR and immunoblotting. CENPE levels were reduced in SF188-miR-137 and UW479-miR-137 cells (Figure 5A) and increased in Res259-anti-miR-137 cells (Figure 5B). KIF14 and NCAPG levels were also decreased in SF188-miR-6500-3p and UW479-miR-6500-3p cells (Figure 5C-5D) as compared to control cells.

We used a 3′UTR luciferase reporter assay to validate the direct targeting of *CENPE*, *KIF14* and *NCAPG* by miR-137 and miR-6500-3p. The putative binding regions of miR-137 and miR-6500-3p within the 3′UTRs of *CENPE*, *KIF14* and *NCAPG* are shown in Figure 5E-5G. Overexpression of miR-137 and of miR-6500-3p suppressed luciferase activity in wild type *CENPE*, *KIF14* or *NCAPG* 3′UTR constructs, but this reduction was rescued when mutated *CENPE*, *KIF14* or *NCAPG* 3′UTR constructs were used (Figure 5E-5G). These findings demonstrated that miR-137 was directly targeted the 3′UTRs of *CENPE*, and miR-6500-3p directly targeted the 3′UTRs of *KIF14* and *NCAPG.*

### *CENPE, KIF14* or *NCAPG* knockdown reduced cell proliferation

**Figure 6:**
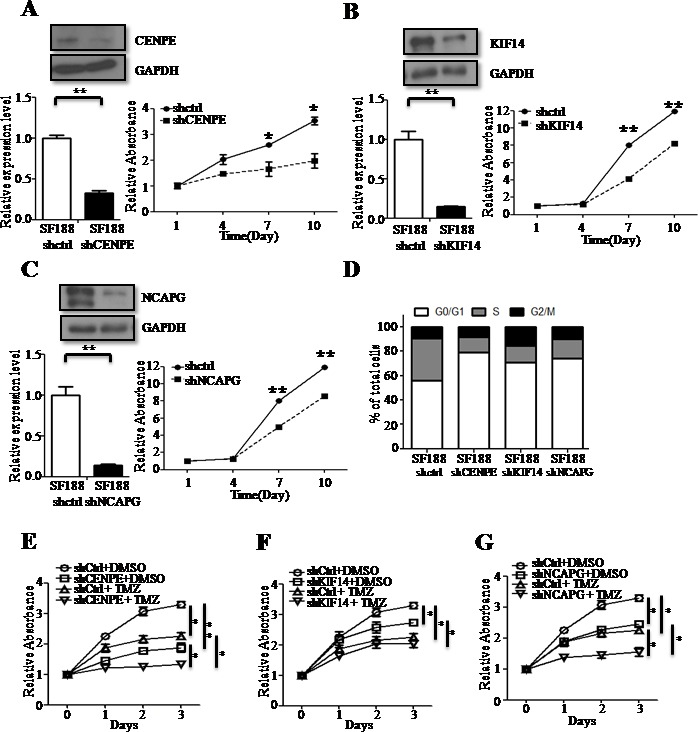
CENPE, KIF14 or NCAPG knockdown (KD)reduces cell proliferation and has a combined anti-proliferative effect in the presence of TMZ CENPE-KD **A.**, KIF14-KD **B.** and NCAPG-KD **C.** decreased cell proliferation rate in SF188 cells asmeasured by MTT assay. KD efficacy was evaluated by RT-qPCR and immunoblotting. CENPE-KD, KIF14-KD and NCAPG-KD SF188 cells were subjected to flow cytometry **D.** CENPE-KD **E.**, KIF14-KD **F.** or NCAPG-KD **G.** SF188 cells were treated with either DMSO or TMZ and cell proliferation was measured at different time points by MTT assay. Results are presented as mean±SD in duplicate samples. *p < 0.05, **p < 0.01 by *t*-test.

The human pHGGs cell lines, SF188 and UW479, were stably transfected with knockdown plasmids targeting *CENPE*, *KIF14* or *NCAPG* as well as a vector control and cell proliferation was measured by MTT assay. Knockdown of *CENPE*, *KIF14* or *NCAPG* resulted in a lower proliferation rate compared to the vector control (Figure 6A-6C & S4A-C). *CENPE*, *KIF14* and *NCAPG* are known to be involved in regulation of the cell cycle. Flow cytometry showed that the knockdown cell lines accumulated cells in the G1 phase of the cell cycle (Figure 6D and S4D). Taken together, our data suggested that *CENPE*, *KIF14* or *NCAPG* knockdown caused cell cycle arrest in G1 phase and impaired cell proliferation.

We then treated *CENPE*, *KIF14* or *NCAPG* knockdown cells with TMZ and measured cell proliferation rates using MTT assay. Proliferation rates were lowest in knockdown cell lines treated with TMZ (TMZ+shCENPE, TMZ+shKIF14 or TMZ+shNCAPG), as compared to control cells (shCtrl), *CENPE, KIF14,* or *NCAPG* knockdown cells without TMZ treatment, or cells treated with TMZ alone (TMZ+shCtrl)(Figure 6E-6F and S4E-F).

## DISCUSSION

Pediatric gliomas are the most common pediatric brain tumors in Taiwan and worldwide. In pediatric CNS tumors identified at Taipei Veterans General Hospital from 1975 to May 2004(patients less than 18 years of age, *n* = 986), the most common tumors were astrocytic, which account for 31.1% of all pediatric CNS tumors [24]. Pediatric HGGs, mainly anaplastic astrocytoma and glioblastoma multiforme, are fast growing and infiltrative malignancies that affect every location within the brain. Despite aggressive surgical resection, radiation and adjuvant chemotherapy treatments, the overall survival rate of pHGG patients is still dismal and much lower than that of pLGG patients (Figure 1A and S1C).

In the present study we showed that pLGGs and pHGGs have unique transcriptome characteristics and that many genes involved in the cell cycle are upregulated in pHGGs. Angiogenesis also plays a crucial role in tumor malignancy and progression, and various angiogenesis-related genes and pathways, especially the EGFR/IGFBP2/HIF2αpathway, are upregulated in pHGGs [25]. Our transcriptome analysis revealed upregulation of a number of angiogenesis-related genes, including *FGF1*, *TGFB2*, *BMP4* and others (*p* < 0.05). Further validations of angiogenesis-related genes through large-scale analyses are still needed.

Dysregulated miRNAs play important roles in the development and progression of a variety of tumors. Therefore, RNA therapies, such as the application of antagomirs or chemically modified miRNAs, have been proposed as potential cancer treatments [26-29]. The present study showed that there are different miRNA signatures between pLGGs and pHGGs. We demonstrated that miR-137 and miR-6500-3p exhibit anti-tumor activity in pediatric glioma cell lines by blocking CENPE, KIF14 or NCAPG expression. miR-137 is reportedly down-regulated in various cancers, including ovarian cancers, melanoma, colorectal cancers, GBM and others. miR-137 overexpression inhibits cell proliferation, migration and angiogenesis by blocking the expression of multiple target genes [14-16, 30-33]. Previously, no study had explored the role of miR-6500-3p in cancer. We identified a number of novel mRNA/miRNA interactions, namely KIF-14/miR-6500-3p and NCAPG/miR-6500-3p, both of which show potential as novel therapeutic strategies for the treatment of pHGGs. Furthermore, we found that only two miRNAs, miR-17-5p and miR-561-5p, were upregulated in pHGGs. miR-17-5p was shown to enhance tumor metastasis and chemoresistance in various cancers, including colon cancers [34], gastric cancers [35], glioblastoma [36] and prostate cancers [37]. However, the biological function of miR-561-5p is currently unknown, and its involvement in pHGG must be elucidated in future work.

In this study, we demonstrated that knockdown of CENPE, KIF14 and NCAPG expression inhibited cell proliferation in pHGGs. Previous studies suggested that *CENPE* acts as a tumor suppressor gene or oncogene in various cancers. CENPE overexpression was correlated with cyclin B1 expression and is related to poor prognosis in breast cancers [38]. Conversely, Liu, *et al.* indicated that CENPE is downregulated in human hepatocellular carcinoma (HCC) tissues [39]. Unlike *CENPE*, *KIF14* acts as an oncogene in various cancers, including lung cancers, ovarian cancers, breast cancers and adult gliomas [40-45]. Previous reports showed that KIF14 knockdown results in cell apoptosis and reduced colony formation [40, 41]. KIF14 overexpression has also been associated with a poor outcome in lung cancers, ovarian cancers, breast cancers and adult gliomas [40-43]. NCAPG in cancers has been less well studied, but it is overexpressed in melanomas [46] and downregulated in out-of-niche primary tumor cells from multiple myelomas and acute myeloid leukemias [47].

This study demonstrated that CENPE, KIF14 or NCAPG knockdown combined with TMZ treatment had a combined suppressive effect on cell proliferation. The CENPE inhibitor, GSK923295, has entered phase I clinical trials and has been found to have only mild side effects in one-third of patients [48]. GSK923295 has been demonstrated to inhibit CENPE motor activity and to cause growth arrest *in vitro* and *in vivo* [49]. Recently, pulsed high-intensity focused ultrasound (HIFU) was demonstrated to temporally disrupt the BBB. A combination of pulsed HIFU and atherosclerotic plaque-specific peptide-1 (AP-1)-conjugated liposomes that contain doxorubicin and that specifically bind to the interleukin 4 receptor (IL-4R) worked to elevate the tumor-to-brain drug ratio in a xenografted GBM model [50-52]. A combination of new therapies, such as GSK923295 and pulsed HIFU, could enhance the effect of TMZ and provide improved pHGG cytoreduction strategies.

In summary, integrated transcriptome analysis demonstrated that cell cycle-related genes, specifically *CENPE*, *KIF14* and *NCAPG*, are upregulated in pHGGs and contribute to cell proliferation. Our study found that *CENPE* expression is inhibited by miR-137, while *KIF14* and *NCAPG* expression are inhibited by miR-6500-3p, and an anti-tumor effect was achieved by combining TMZ with either miR137 or miR-6500-3p. The unique transcriptomes and miRNomes of pHGGs and pLGGs analyzed in this study provide insight into the mechanisms of pediatric gliomagenesis and could aid in the development of new anti-tumor therapeutic strategies.

## MATERIALS AND METHODS

### Biological samples

This study was approved by the Institutional Ethics Committee/Institutional Review Board of Taipei Veterans General Hospital (VGH-TPE). The parents/legal guardians of patients in this study provided informed consent (VGHIRB No.: 2013-07-016A, 2013-01-020B&2014-05-006C). Fresh tumor tissues were removed during surgery and then snap-frozen, followed by storage in liquid nitrogen until RNA extraction. Overall survival time was calculated as the time from diagnosis or surgery until death or last follow-up appointment. Differences in survival times were assessed using the log-rank test.

### Cell cultures and plasmids

Pediatric glioma cell lines used in this study were kindly provided by Pediatric Oncology, the Institute of Cancer Research, Sutton, United Kingdom [53], and included Res259 (pediatric astrocytoma, Grade II), UW479 (pediatric anaplastic astrocytoma, Grade III) and SF188 (pediatric glioblastoma multiforme, Grade IV). Glioma cells and embryonic kidney 293T cells were cultured in Dulbecco's Modified Eagle Medium-F12 (DMEM-F12) and Dulbecco's Modified Eagle Medium (DMEM)(GIBCO, Grand Island, NY, USA) supplemented with 10% fetal bovine serum (FBS) (GIBCO) in 5% CO_2_ at 37°C, respectively.

shCENPE, shKIF14, shNCAPG and a control plasmid were obtained from the RNAi consortium at Academia Sinica, Taiwan. For miRNA expression using a plasmid, human miR-137 and miR-6500-3p were PCR-amplified from genomic DNA (Table S3) and cloned into the pLenti4 expression vector (Invitrogen). miRZip-137, which allows for stable expression of anti-microRNA-137(a lentivirus-based plasmid), was purchased from System Biosciences (SBI). All 3′UTR reporter plasmids were PCR-amplified from human genomic DNA (Table S3) and cloned into the pGL3-Basic reporter plasmid (Promega).

### Microarray and computational analyses

The Affymetrix^TM^ HG-U133 Plus 2.0 whole genome array was applied as described previously [54]. Principal component analysis (PCA) was performed using Partek Genomics Suite software (http://www.partek.com). A Gene Ontology database search was performed using Ingenuity Pathway Analysis (http://www.ingenuity.com/products/ipa).

### Small RNA sequencing (smRNA-Seq) and data analysis

Total RNA sample preparation, sequencing and data analysis were performed as described previously [55]. Briefly, total RNA was collected and small RNA fractions were sequenced using an Illumina HiSeq2000 sequencer (Illumina, San Diego, CA USA) according to manufacturer's instructions. Raw sequencing reads in fastq format were subjected to our in-house bioinformatics pipelines for miRNA profiling and discovery [13].

### Quantitative real-time reverse transcription-PCR (qRT-PCR)

Total RNA was isolated from fresh-frozen tumor samples and cells using the DNeasy Blood & Tissue and RNAeasy Kits, respectively (Qiagen, GmbH, Germany). Next, 1 μg of total RNA was used to perform reverse transcription (RT) with a RevertAid™ Reverse transcriptase kit (Cat. K1622; Fermentas, Glen Burnie, Maryland, USA). For qPCR, paired-primers were used to detect specific genes and miRNAs (Table S3).

### Immunoblotting

Knockdown or miRNA expression plasmids were introduced into pediatric glioma cell lines using lentiviruses. After 48 h, cells were harvested and lysed using NET lysis buffer containing protease and phosphatase inhibitors. Immunoblotting was carried out using anti-CENPE (1:1000, OriGene Technologies), anti-KIF14 (1:5000, Sigma-Aldrich) and anti-NCAPG (1:5000, Sigma-Aldrich) antibodies, followed by visualization with horseradish peroxidase-conjugated secondary antibodies and an enhanced chemiluminescence detection system (Millipore).

### Proliferation assay

Pediatric glioma cells were seeded in 24-well plates at 2×10^3^ cells per well. After 1, 4, 7 and 10 days, cells were treated with 1 ml of 3-(4,5-dimethylthiazol-2-yl)-2,5-diphenyltetrazolium bromide (MTT) in phosphate buffered saline (PBS) for 2 h. Purple formazan was solubilized in0.5 ml isopropanol containing 0.1% sodium dodecyl sulfate (SDS). One-hundred μl of solution was transferred into a 96-well plate and optical density was measured by a spectrophotometer at 570 nm.

### Cell cycle analysis

5×10^5^ cells were fixed in cold 70% alcohol overnight at −20°C, and then treated with 0.1% TritonX-100 containing 0.2 mg/ml RNase for 1.5 hat 37°C. Cells were then washed with PBS, stained with propidium iodide (PI) for 30 min and analyzed on a FACSCanto (BD Pharmingen). Results were processed using FlowJo.

### *In vitro* drug sensitivity assay

Pediatric glioma cells were seeded at 5×10^4^ cells per well in DMEM-F12 supplemented with 10% FBS into a 24-well plate. After 24 h, medium was removed and new medium containing 250 μM temozolomide (CAS_No. 85622931; Sigma) was added to the test group (day 0). After drug treatment for various times (24, 48, 72h), cells were incubated with 500μl of 0.1 mg/ml MTT in PBS for 2 h. Purple formazan was then solubilized using 1 ml of isopropanol containing 0.1% sodium dodecyl sulfate (SDS) and the optical density was measured by spectrophotometer at 570 nm.

### Reporter assay

The microRNA expression plasmids (500 ng), the reporter plasmid (100 ng), and the renilla luciferase plasmid (10 ng) were co-transfected into 1×10^5^ HEK293T cells using the TurboFect transfection reagent (Thermo Scientific). Luciferase activity was measured using a luminometer (model LB593; Berthod, Bad Wildbad, Germany). All assays were performed in duplicate and transfection experiments were repeated at least three times. Firefly luciferase activity was normalized against Renilla luciferase activity.

### Immunohistochemistry

IHC sample preparation and staining were performed as described previously [54]. Antibodies used were anti-CENPE (1:500, Sigma-Aldrich), anti-KIF14 (1:200, Sigma-Aldrich) and anti-NCAPG (1:50, Sigma-Aldrich).

### Statistical analysis

Two-tailed Student's t-testswere used to assess the significance of mean differences. Differences were considered significant at a *p*≤0.05. The relationships of microRNAs and their target genes were evaluated with Pearson's correlation coefficients *r.*

## SUPPLEMENTARY MATERIAL FIGURES AND TABLES








