# Supplementary material for: Downregulation of miR-137 and miR-6500-3p promotes cell proliferation in pediatric high-grade gliomas
Source: Oncotarget. 2016 Feb 25;7(15):19723–37. doi: 10.18632/oncotarget.7736 (PMC4991414; doi:10.18632/oncotarget.7736)
Supplement: Supplementary file 1 [file oncotarget-07-19723-s001.pdf]

Downregulation of miR-137 and miR-6500-3p promotes cell proliferation in pediatric high-grade gliomas

Supplementary Material

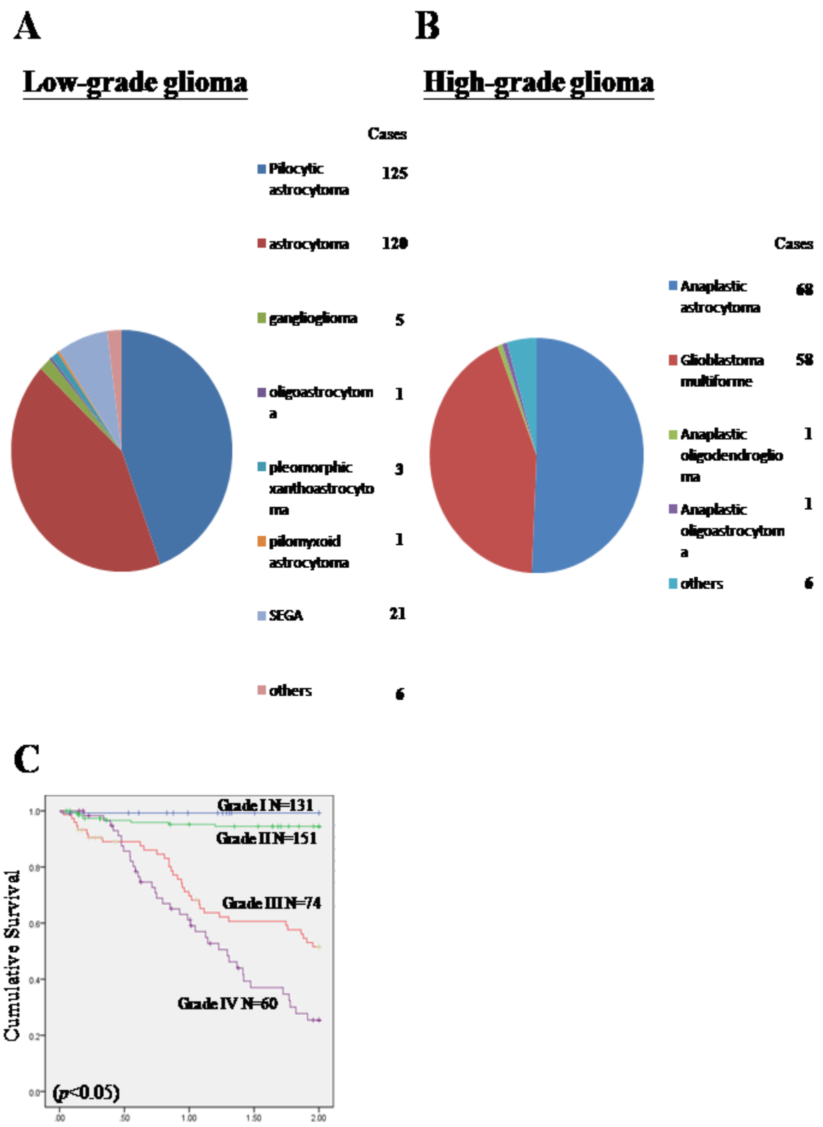

Supplementary Figure 1: Subtype case numbers for pLGG A. and pHGG B. and2-year Kaplan-Meier survival curves for pediatric glioma patients at Taipei Veterans General Hospital between 1971 and 2013 C. Blue line: grade I (LGG); green line: grade II (LGG); red line: grade III (HGG); purple line: grade IV (HGG).

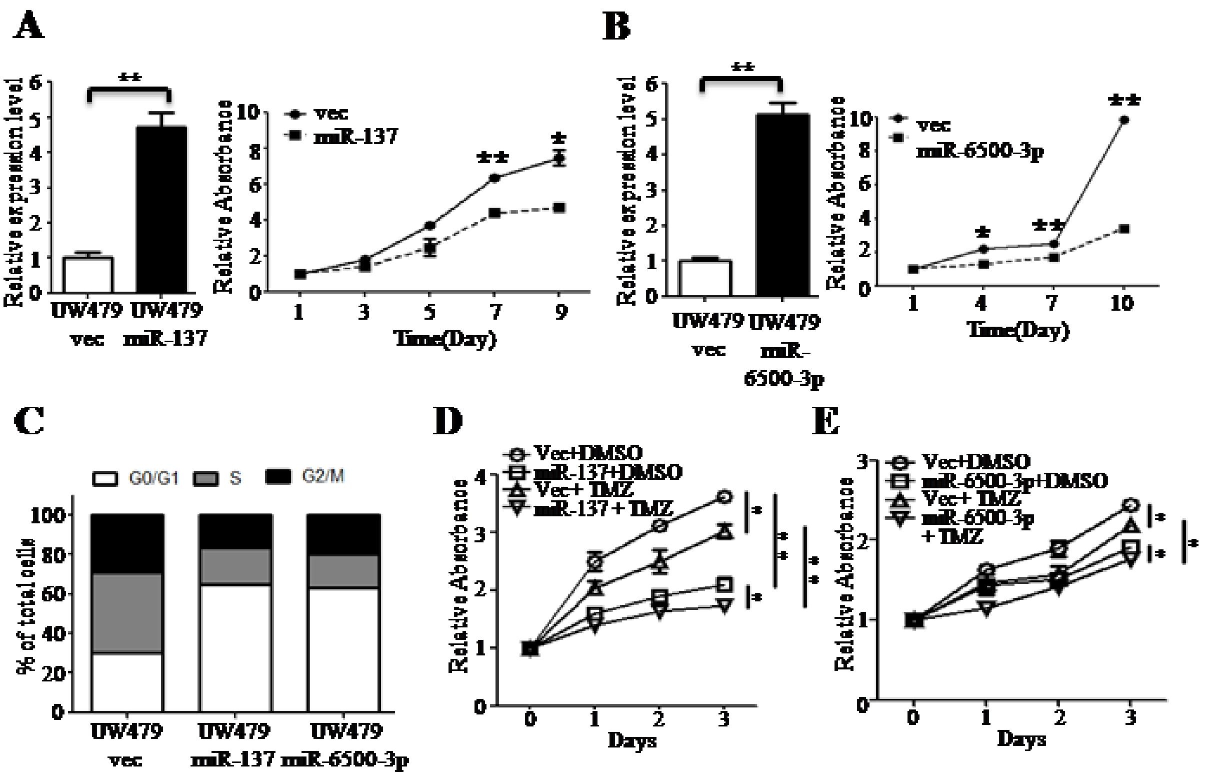

Supplementary Figure 2: miR-137 and miR-6500-3p suppress cell proliferation and have a combined effect with TMZ treatment. Overexpression of miR-137 **A.**ormiR-6500-3p **B.** decreased cell proliferation in UW479 cells as measured by MTT assay. Results were presented as mean±SD for duplicate samples. *p<0.05, **p<0.01 by t-test. The efficiency of overexpressed was evaluated by RT-qPCR. UW479 cells overexpressing either miR-137 or miR-6500-3p were subjected to flow cytometry**C.**UW479 cells overexpressing either miR-137 **D.** ormiR-6500-3p **E.** were treated with either DMSO or TMZ and cell proliferation was measured at different time points by MTT assay. Results are presented as mean±SD for duplicate samples. *p<0.05, **p<0.01 by t-test.

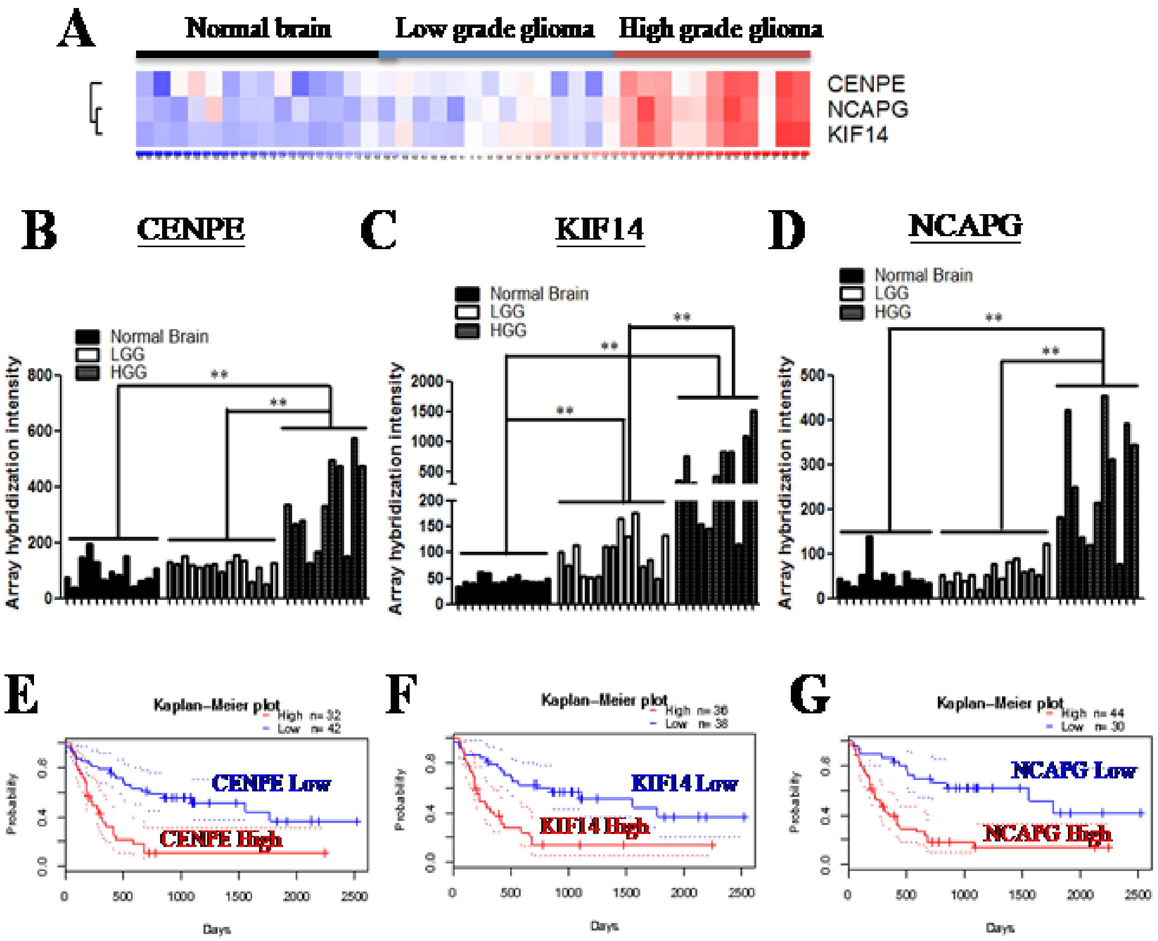

Supplementary Figure 3:CENPE, KIF14 and NCAPG expression measured by microarray analysis of pediatric gliomas obtained from another study. Heat map representing CENPE, KIF14 and NCAPG expression (q<0.01) from 13 normal brain tissue samples, 15 pLGG samples and 11 pHGG samples **A.** CENPE **B.**, KIF14 **C.** and NCAPG **D.** array hybridization signals. *p<0.05, **p<0.01 by t-test. Prognoscan showed prognosis results for CENPE **E.**, KIF14 **F.** and NCAPG **G.**

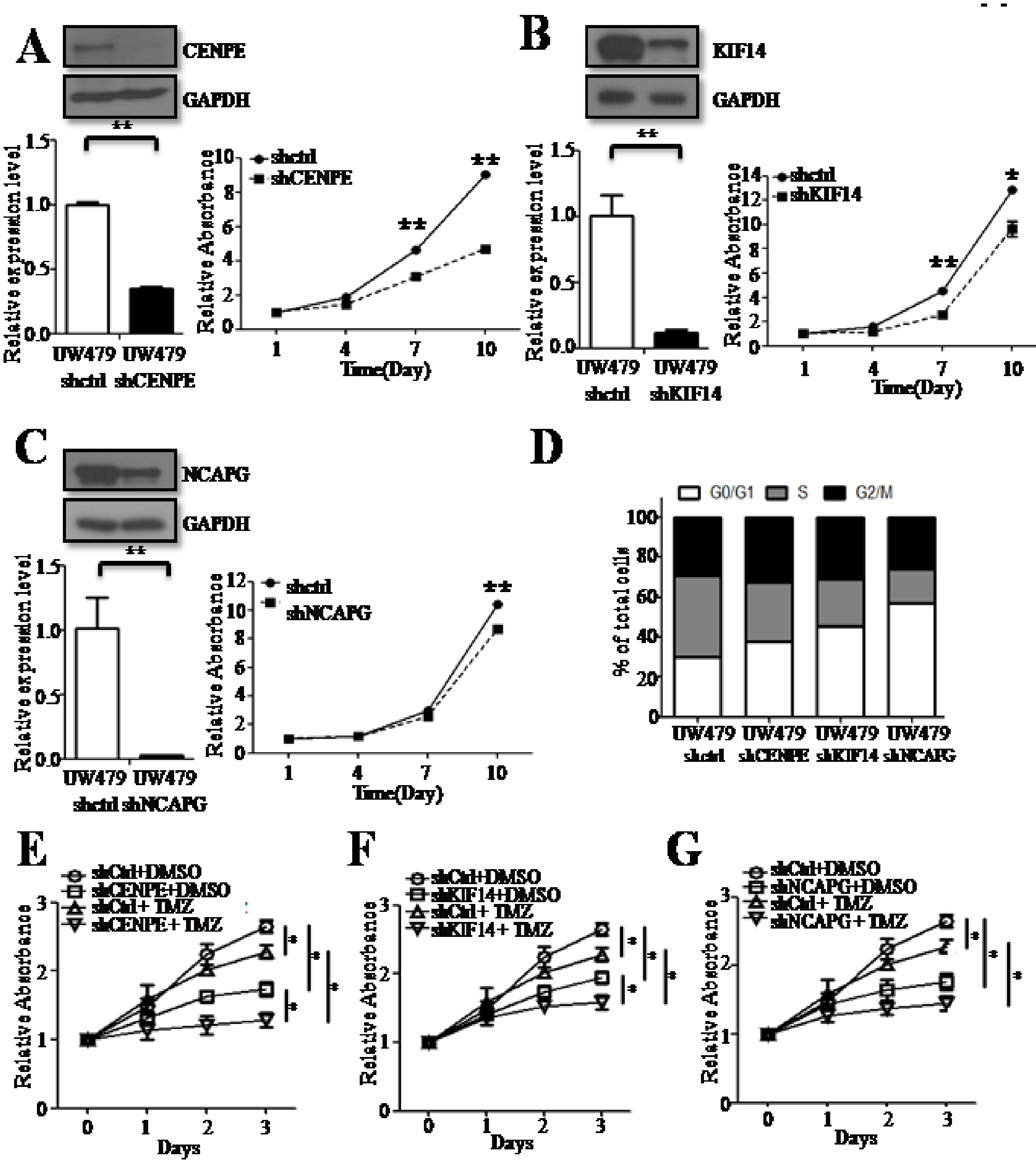

Supplementary Figure 4:CENPE, KIF14 or NCAPG knockdown (KD) reduced cell proliferation and had a combined anti-proliferative effect in the presence of temozolomide. Knockdown of CENPE **A.**, KIF14 **B.** or NCAPG **C.** decreases cell proliferation in UW479 cells as measured by MTT assay. Knockdown efficiency was evaluated by RT-qPCR and

immunoblotting. CENPE-KD, KIF14-KD and NCAPG-KD UW479 cells were subjected to flow cytometry assay **D.** CENPE-KD **E.**, KIF14-KD **F.** or NCAPG-KD **G.** UW479 cells were treated with either DMSO or TMZ and cell proliferation was measured at different time points by MTT assay. Results were presented as mean±SD for duplicate samples. **p*<0.05, ***p*<0.01 by t-test.
